# A 3D Hologram With Mixed Reality Techniques to Improve Understanding of Pulmonary Lesions Caused by COVID-19: Randomized Controlled Trial

**DOI:** 10.2196/24081

**Published:** 2021-09-10

**Authors:** Songxiang Liu, Mao Xie, Zhicai Zhang, Xinghuo Wu, Fei Gao, Lin Lu, Jiayao Zhang, Yi Xie, Fan Yang, Zhewei Ye

**Affiliations:** 1 Department of Orthopedics Union Hospital, Tongji Medical College Huazhong University of Science and Technology Wuhan China; 2 Intelligent Medical Laboratory Union Hospital, Tongji Medical College Huazhong University of Science and Technology Wuhan China; 3 Department of Radiology Union Hospital, Tongji Medical College Huazhong University of Science and Technology Wuhan China

**Keywords:** COVID-19, mixed reality, hologram, pulmonary, lesion, diagnostic, imaging

## Abstract

**Background:**

The COVID-19 outbreak has now become a pandemic and has had a serious adverse impact on global public health. The effect of COVID-19 on the lungs can be determined through 2D computed tomography (CT) imaging, which requires a high level of spatial imagination on the part of the medical provider.

**Objective:**

The purpose of this study is to determine whether viewing a 3D hologram with mixed reality techniques can improve medical professionals’ understanding of the pulmonary lesions caused by COVID-19.

**Methods:**

The study involved 60 participants, including 20 radiologists, 20 surgeons, and 20 medical students. Each of the three groups was randomly divided into two groups, either the 2D CT group (n=30; mean age 29 years [range 19-38 years]; males=20) or the 3D holographic group (n=30; mean age 30 years [range 20=38 years]; males=20). The two groups completed the same task, which involved identifying lung lesions caused by COVID-19 for 6 cases using a 2D CT or 3D hologram. Finally, an independent radiology professor rated the participants' performance (out of 100). All participants in two groups completed a Likert scale questionnaire regarding the educational utility and efficiency of 3D holograms. The National Aeronautics and Space Administration Task Load Index (NASA-TLX) was completed by all participants.

**Results:**

The mean task score of the 3D hologram group (mean 91.98, SD 2.45) was significantly higher than that of the 2D CT group (mean 74.09, SD 7.59; *P*<.001). With the help of 3D holograms, surgeons and medical students achieved the same score as radiologists and made obvious progress in identifying pulmonary lesions caused by COVID-19. The Likert scale questionnaire results showed that the 3D hologram group had superior results compared to the 2D CT group (teaching: 2D CT group median 2, IQR 1-2 versus 3D group median 5, IQR 5-5; *P*<.001; understanding and communicating: 2D CT group median 1, IQR 1-1 versus 3D group median 5, IQR 5-5; *P*<.001; increasing interest: 2D CT group median 2, IQR 2-2 versus 3D group median 5, IQR 5-5; *P*<.001; lowering the learning curve: 2D CT group median 2, IQR 1-2 versus 3D group median 4, IQR 4-5; *P*<.001; spatial awareness: 2D CT group median 2, IQR 1-2 versus 3D group median 5, IQR 5-5; *P*<.001; learning: 2D CT group median 3, IQR 2-3 versus 3D group median 5, IQR 5-5; *P*<.001). The 3D group scored significantly lower than the 2D CT group for the “mental,” “temporal,” “performance,” and “frustration” subscales on the NASA-TLX.

**Conclusions:**

A 3D hologram with mixed reality techniques can be used to help medical professionals, especially medical students and newly hired doctors, better identify pulmonary lesions caused by COVID-19. It can be used in medical education to improve spatial awareness, increase interest, improve understandability, and lower the learning curve.

**Trial Registration:**

Chinese Clinical Trial Registry ChiCTR2100045845; http://www.chictr.org.cn/showprojen.aspx?proj=125761

## Introduction

The COVID-19 outbreak has now become a pandemic [1]. It has had a serious adverse impact on global public health [2]. Many doctors are on the front line of fighting the epidemic, including orthopedic surgeons, general surgeons, and neurosurgeons. Two-dimensional computed tomography (CT) scanning, which requires high spatial imagination on the part of medical professionals, has been traditionally used to determine the status of a pulmonary infection [3,4]. Often, judging the specific condition of the pulmonary lesions caused by COVID-19 would require the expertise of experienced respiratory, radiology, infectious disease, and intensive care department experts. Doctors who are not specialized in respiratory-related cases, not to mention medical students and the public, may not find it easy to understand the clinical significance of a 2D CT scan.

Both doctors and the public have played a vital role in combating COVID-19. Doctors treat patients with COVID-19 and fight the virus directly. The public's self-protection and isolation efforts help to stop the spread of the virus. However, there are still many members of the public who are not aware of the necessary protective measures. In addition, it is very important for medical students to have a better understanding of COVID-19, as they are the successors in the fight against this virus. This dilemma may be experienced by other countries as well.

Mixed reality techniques can overlap virtual and real worlds. The user can view the real world while manipulating the digital content generated by a device [5]. Mixed reality has been used clinically to help doctors better understand anatomical structures [6-8]. However, the use of 3D holograms to image lungs affected by COVID-19 has not been reported in the literature. Therefore, we present a new method that applies mixed reality techniques to create a 3D hologram of lungs affected by COVID-19.

## Methods

### Trial Design

The study was a parallel-group randomized controlled trial. It was conducted in Wuhan, China, from March 2020 to September 2020. Participants were randomized into two groups in a 1:1 ratio: (1) a 3D holographic intervention group or (2) a 2D CT control group.

### Participants and Recruitment

We randomly selected radiologists, surgeons, and medical students from our institution and asked them to participate in this study. The radiologists, surgeons, and medical students were eligible to participate in this trial if they had not previously seen 3D holograms of lungs affected by COVID-19. The study involved 60 participants, including 20 radiologists, 20 surgeons, and 20 medical students. Each of the three groups was randomly divided into two groups: (1) the 2D CT group and (2) the 3D holographic group (Figure S1 in Multimedia Appendix 1). Exclusion criteria were as follows: surgeons and medical students trained in COVID-19 lung CT imaging, and participants that had previously seen 3D holograms of COVID-19 lungs.

### Intervention

For this study, 3D reconstructions of patients’ lungs were made using data from CT scans using a StarCloud workstation (a 3D reconstruction software from Visual3D). Polygon mesh files were exported from the StarCloud workstation. These were uploaded into the StarCloud Mixed Reality system (Visual3D). After uploading the data, 3D holographic images were automatically converted as a case-specific computer graphic for mixed reality, referred to as a hologram. The data pertaining to the hologram can be downloaded onto a Microsoft HoloLens (Microsoft Corp).

The two groups completed the same task, which involved identifying lesion areas in the lungs of 6 cases of COVID-19 using a 2D CT or 3D hologram of the lungs. The 6 cases of COVID-19 used for the study involved moderate to severe COVID-19 (Table S1 in Multimedia Appendix 1).

### Assessments

An independent radiology professor rated the participants' performance (out of 100). All participants in the two groups completed a Likert scale questionnaire regarding the educational utility and efficiency of 3D holograms. The National Aeronautics and Space Administration Task Load Index (NASA-TLX) was completed by all participants. The NASA-TLX is a multidimensional rating procedure that provides an overall workload score between 0 and 100 based on a weighted average of ratings on 6 subscales [9]: (1) mental demands (“How mentally demanding was the task?”), (2) physical demands (“How physically demanding was the task?”), (3) temporal demands (“How hurried or rushed was the pace of the task?”), (4) own performance (“How successful were you in performing the task?”), (5) effort (“How hard did you have to work to achieve your level of performance?”), and (6) frustration (“How insecure, discouraged, irritated, stressed, and annoyed were you?”). The ethics committee of Wuhan Union Hospital, Tongji Medical College, Huazhong University of Science and Technology approved this study, and all participants provided signed informed consent.

### Statistical Analysis

Statistical analyses were performed using SPSS (version 19; IBM Corp). Continuous variables were expressed as means and standard deviations. Results of the NASA-TLX questionnaires were summarized in terms of means and standard deviations. Data were processed using analysis of variance to determine possible relationships between individual characteristics and workload.

## Results

### Sample Characteristics

Participant baseline characteristics are described in Table 1. The study involved 60 participants, including 20 radiologists, 20 surgeons, and 20 medical students. Each of the three groups was randomly divided into two groups: (1) the 2D CT group (n=30; mean age 29 years [range 19-38 years]; males=20) and (2) the 3D holographic group (n=30; mean age 30 years [range 20-38 years]; males=20; Figure S1 in Multimedia Appendix 1).

**Table 1:** Demographic information.

Variables		3D holographic group (n=30)	2D computed tomography group (n=30)
**Gender**			
	Male	20	20
	Female	10	10
Age (year), mean (range)		30 (20-38)	29 (19-38)
**Participants**			
	Radiologists, total (male)	10 (5)	10 (5)
	Surgeons, total (male)	10 (10)	10 (10)
	Medical students, total (male)	10 (5)	10 (5)

### Outcomes

**Figure 1:**
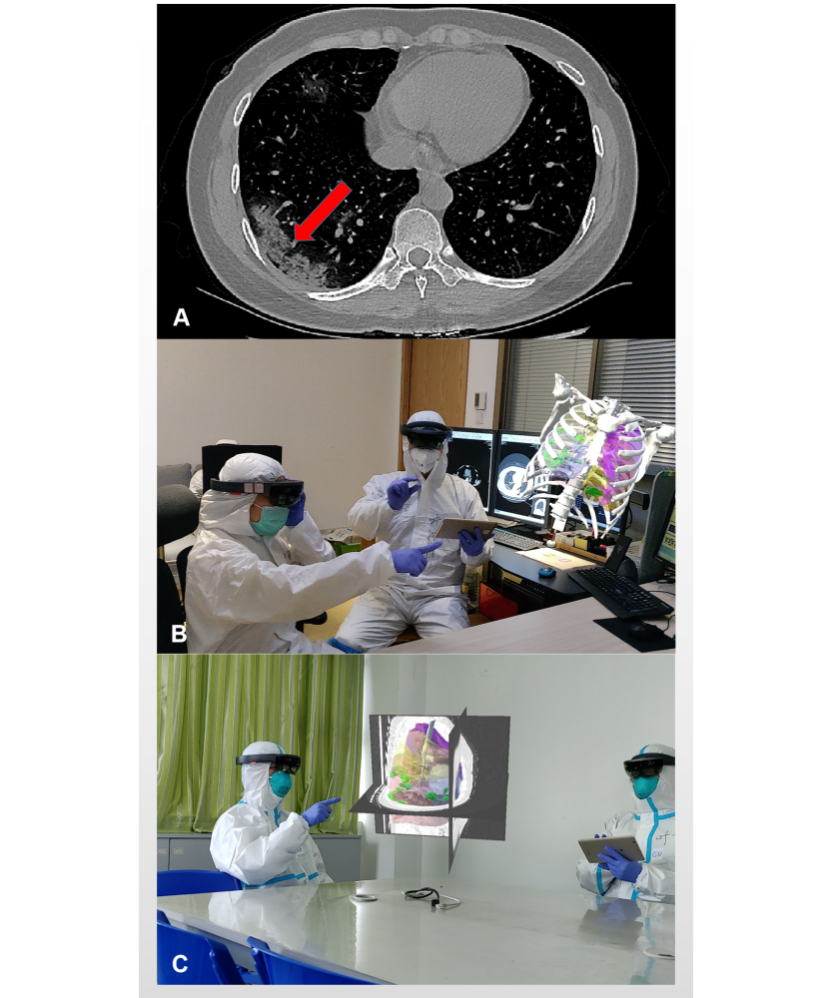
A 3D hologram of the pulmonary lesions caused by COVID-19. (A) A computed tomography scan of a patient with COVID-19 revealed patchy pure ground-glass opacities (red arrow). (B) A hologram can clearly show the lesion site and lesion range (green color) of the patient's lung infection, allowing stereoscopic viewing from 360 degrees in a physical world. (C) Simultaneous analysis of 2D and 3D images.

**Figure 2:**
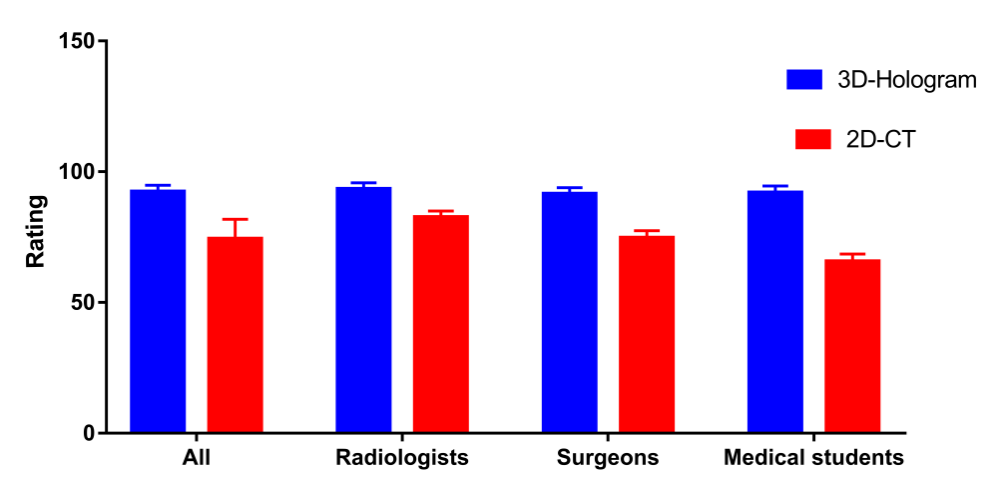
Task scores for identifying COVID-19 lesion areas in lungs. In the mixed reality group, the mean task score was significantly higher than that of the conventional group, with *P*<.001. With the help of 3D holograms, surgeons and medical students achieved the same score as radiologists and made obvious progress in identifying the pulmonary lesions caused by COVID-19.

**Figure 3:**
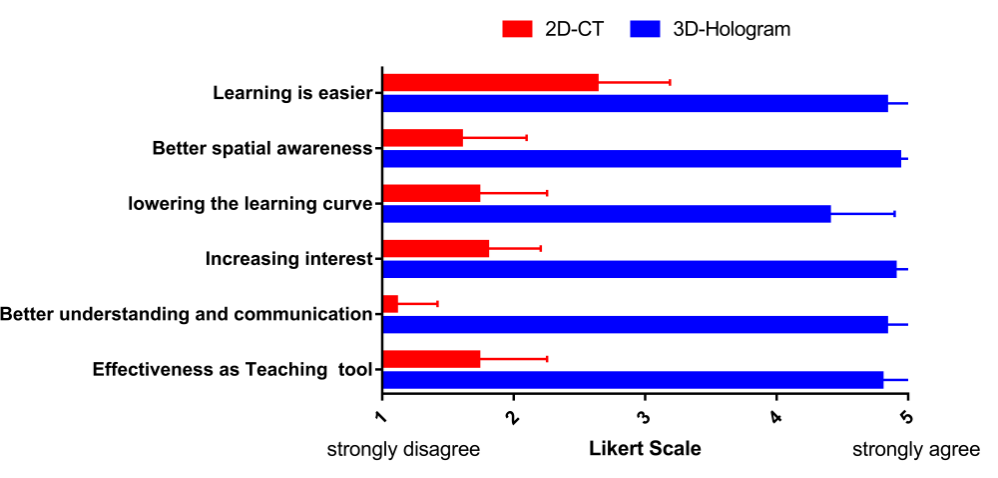
Responses to a Likert scale questionnaire regarding the educational utility and efficiency of 3D holograms. The 3D hologram group indicated higher educational utility and efficiency than the 2D CT group. CT: computed tomography.

Clear 3D visual holographic renderings were obtained for the lungs of patients with COVID-19 (Figure 1). In the 3D holographic (mixed reality) group, the task scores for all participants (mean 91.98, SD 2.45), radiologists (mean 93.60, SD 2.25), surgeons (mean 91.50, SD 2.31), and medical students (mean 91.25, SD 2.18) were significantly higher than that of the conventional (2D) group (all participants: mean 74.09, SD 7.59; radiologists: mean 82.63, SD 2.28; surgeons: mean 74.55, SD 2.52; medical students: mean 65.10, SD 65.10; *P*<.001). With the help of 3D holograms, surgeons and medical students achieved the same scores as radiologists and made obvious progress in identifying pulmonary lesions caused by COVID-19 (Figure 2, Table 2). The Likert scale questionnaire revealed that the 3D hologram group had superior results compared to the 2D CT group (teaching: 2D CT group median score 2, IQR 1-2 versus 3D group median 5, IQR 5-5; *P*<.001; understanding and communicating: 2D CT group median score 1, IQR 1-1 versus 3D group median 5, IQR 5-5; *P*<.001; increasing interest: 2D CT group median score 2, IQR 2-2 versus 3D group median 5, IQR 5-5; *P*<.001; lowering the learning curve: 2D CT group median score 2, IQR 1-2 versus 3D group median 4, IQR 4-5; *P*<.001; spatial awareness: 2D CT group median score 2, IQR 1-2 versus 3D group median 5, IQR 5-5; *P*<.001; learning: 2D CT group median score 3, IQR 2-3 versus 3D group median 5, IQR 5-5; *P*<.001; Figure 3, Table 2).

**Figure 4:**
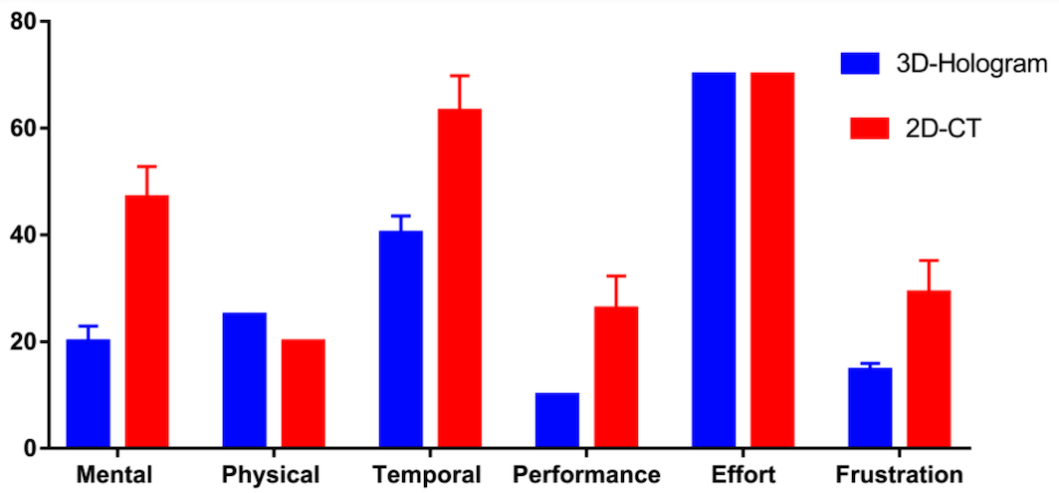
Results from the National Aeronautics and Space Administration (NASA) Task Load Index assessment. The mean rating for each subscale is given. Those that used holograms scored significantly lower than those that used 2D CT scans for task load on the "mental," "temporal," "performance," and "frustration" subscales. CT: computed tomography.

The 3D hologram group scored significantly lower than the 2D CT group on the “mental,” “temporal,” “performance,” and “frustration” subscales of the NASA-TLX. Compared with the traditional 2D group, the 3D hologram group reported higher physical demands. The main reason is that participants need to wear a mixed reality headset, but the weight of the mixed reality glasses was acceptable (Figure 4, Table 2).

**Table 2:** Task scores, NASA Task Load Index scores, and Likert scale questionnaire scores.

Category		2D CT^a^ group, mean (SD)	2D CT group, median (IQR)	3D holographic group, mean (SD)	3D holographic group, median (IQR)	*P* value
**Task score**						
	All participants	74.09 (7.59)	75 (68-80)	91.98 (2.45)	90 (90-95)	<.001
	Radiologists	82.63 (2.28)	83 (80-85)	93.60 (2.25)	95 (90-95)	<.001
	Surgeons	74.55 (2.52)	75 (75-75)	91.50 (2.31)	90 (90-95)	<.001
	Medical students	65.10 (2.61)	65 (65-68)	91.25 (2.18)	90 (90-94)	<.001
**NASA Task Load Index scores**						
	Mental	47.03 (5.71)	50 (40-50)	20.06 (2.85)	20 (20-20)	<.001
	Physical	19.75 (1.09)	20 (20-20)	25.08 (0.64)	25 (25-25)	<.001
	Temporal	63.11 (6.45)	60 (60-70)	40.31 (3.04)	40 (40-40)	<.001
	Performance	26.17 (6.03)	25 (20-30)	10.08 (0.99)	10 (10-10)	<.001
	Effort	69.92 (0.99)	70 (70-70)	69.92 (0.99)	70 (70-70)	.99
	Frustration	29.22 (5.95)	30 (25-35)	14.58 (1.39)	15 (15-15)	<.001
**Likert-scale questionnaire scores**						
	Effectiveness as teaching tool	1.73 (0.51)	2 (1-2)	4.8 (0.40)	5 (5-5)	<.001
	Better understanding and communication	1.1 (0.30)	1 (1-1)	4.83 (0.37)	5 (5-5)	<.001
	Increasing interest	1.8 (0.4)	2 (2-2)	4.9 (0.3)	5 (5-5)	<.001
	Lowering the learning curve	1.73 (0.51)	2 (1-2)	4.4 (0.49)	4 (4-5)	<.001
	Better spatial awareness	1.6 (0.49)	2 (1-2)	4.93 (0.25)	5 (5-5)	<.001
	Learning is easier	2.63 (0.55)	3 (2-3)	4.83 (0.37)	5 (5-5)	<.001

^a^CT: computed tomography.

## Discussion

A 3D hologram with mixed reality techniques can be used to help medical professionals, especially medical students and newly hired doctors, better identify pulmonary lesions caused by COVID-19. It can be used in medical education to increase interest, improve understandability, improve spatial awareness, and lower the learning curve.

A CT scan of the lungs of patients with COVID-19 revealed patchy pure ground-glass opacities (Figure 1A). We visualized the patients’ lungs using mixed-reality technology, which can transform the 2D CT image of the lung into a 3D hologram (Figure 1B) to clearly show the lesion site and lesion range (green color) of the patient’s lung infection, allowing stereoscopic viewing from 360 degrees in a physical world. Conversely, a standard 2D CT usually only allows one to see the local area of a certain layer of the lung. The 3D hologram can also analyze 2D and 3D images simultaneously (Figure 1C). As the 3D hologram can clearly show the spatial anatomical neighborhood, it does not require the human brain to mentally transform the complex 2D structure into a complete 3D structure. The results of this study showed that in the 3D hologram group, compared with the traditional 2D CT group, task loads for the “mental,” “temporal,” and “frustration” subscales were significantly reduced, and better performance was obtained. Thus, this study can help doctors—whether they have just started to practice, are in nonrespiratory specialties, or are in respiratory specialties—better identify and understand the pulmonary lesions caused by COVID-19. In addition, this can improve medical students’ understanding of COVID-19, which is important as they are the successors in the fight against this virus.

**Figure 5:**
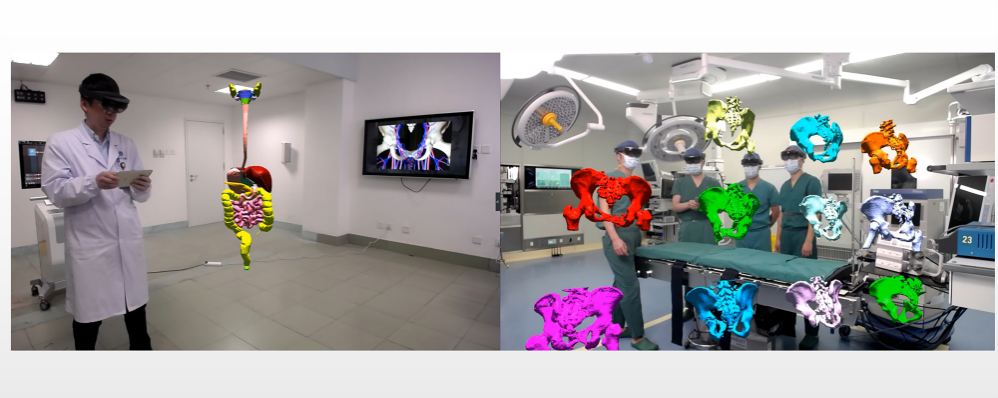
Images show a 3D holographic mixed reality technology being used to present and teach the digestive system and classification of pelvic fractures.

This technique of image reconstruction and presentation can also be applied to other anatomical systems throughout the body (Figure 5). It can contribute to a better understanding of normal and abnormal body structure for both medical and nonmedical individuals and could be especially useful for medical students when used in future medical education. In addition, unlike traditional 3D anatomical drawings, mixed reality technology allows multiple people to view the same 3D hologram from 360 degrees during the teaching process.

The COVID-19 outbreak has also created challenges for anatomy education [10]. Cadaver specimens were the standard learning method of anatomy classes in the past [11]. However, substantial financial, ethical, and supervisory constraints on their use and the shortage of cadaver specimens are ongoing problems faced by teaching colleges and universities [12]; in addition, during the COVID-19 epidemic, the use of cadaver specimens has the potential risk of virus transmission [10,13]. Digital anatomical imaging is a feasible alternative solution to the use of cadaver specimens and is more visual, accessible, clean, fun, and inexpensive.

Although this study shows promising results, further studies in more institutions, populations, and locations are needed in the future.

In conclusion, a 3D hologram with mixed reality techniques can provide a better understanding of the pulmonary lesions caused by COVID-19 and will play an important role in future medical education.

## Appendix

Multimedia Appendix 1 Information about computed tomography images of COVID-19 lungs.

Multimedia Appendix 2 CONSORT-eHEALTH checklist V 1.6.1.

## Abbreviations

CT: computed tomography
NASA-TLX: National Aeronautics and Space Administration Task Load Index
