# Supplementary material for: A 3D Hologram With Mixed Reality Techniques to Improve Understanding of Pulmonary Lesions Caused by COVID-19: Randomized Controlled Trial
Source: J Med Internet Res. 2021 Sep 10;23(9):e24081. doi: 10.2196/24081 (PMC8437403; doi:10.2196/24081)
Supplement: Multimedia Appendix 1 [file jmir_v23i9e24081_app1.doc]

**Figure S1.** Flowchart of the pilot study

**60** participants

**20** radiologists, **20** surgeons, **20** medical students

(10 men) (20 men) (10 men)

Each group was randomly assigned according to gender

**2D CT group**

**30** participants(20men)

**10** radiologists(5men)

**20** surgeons(10men)

**20** medical students(5men)

**3D holographic group**

**30** participants(20men)

**10** radiologists(5men)

**20** surgeons(10men)

**20** medical students(5men)

| **Table S1 The information of CT images of COVID19** | | | | | |
| --- | --- | --- | --- | --- | --- |
| **Case number** | **Gender** | **Age(year)** | **Extent cm^3^ (%)** | **location** | **characteristics** |
| 1 | Male | 72 | 1679.05  (41.97%) | The whole lung | GGO /Consolidation |
| 2 | Female | 61 | 816.67  (35.74%) | The whole lung | GGO /Consolidation |
| 3 | Female | 37 | 210.78  (5.37%) | The whole lung | GGO |
| 4 | Female | 68 | 189.6  (6.87%) | Rt. middle/ lower/ Lt. upper lobe | GGO /Consolidation |
| 5 | Male | 61 | 1004.79  (42.01%) | The whole lung | GGO /Consolidation |
| 6 | Female | 56 | 450.93  (15.36%) | The whole lung | GGO /Consolidation |
